# The Effect of Positive Therapeutic Communication on Pain (POPAIN) and Anxiety During Arterial Blood Gas Standardized Procedures in the Emergency Department Compared to Traditional Communication: Protocol for a Monocentric Randomized Controlled Trial

**DOI:** 10.2196/42043

**Published:** 2023-06-13

**Authors:** Thomas Schmutz, Christophe Le Terrier, Vincent Ribordy, Katia Iglesias, Youcef Guechi

**Affiliations:** 1 Department of Emergency Medicine Fribourg Hospital Fribourg Faculty of Medicine, Fribourg University Fribourg Switzerland; 2 Division of Intensive Care Geneva University Hospitals University of Geneva Geneva Switzerland; 3 School of Health Sciences HES-SO University of Applied Sciences and Arts of Western Switzerland Fribourg Switzerland

**Keywords:** pain, therapeutic communication, nocebo, emergency department, arterial blood gas, anxiety, positive therapeutic communication

## Abstract

**Background:**

In the emergency department (ED), medical procedures, such as arterial blood gas (ABG) testing, can cause pain and high stress levels. However, ABG testing is a routine procedure assessing the severity of the patient's condition. To reduce the pain of ABG, several methods have been investigated without significant difference in pain perception. Communication, a key element of care, has shown a significant effect on pain perception. A positive communication strategy, including positive, kind, or reassuring words, can reduce pain perception, while negative words can raise this perception, causing discomfort, known as the “nocebo effect.” Although some studies have compared the impact of verbal attitudes, particularly in anesthesia and mainly with staff already trained in hypnosis, to the best of our knowledge, none have investigated the effect of communication in the emergency setting, where patients may be more suggestible to the words used.

**Objective:**

In this study, we will investigate the effect of positive therapeutic communication on pain, anxiety, discomfort, and global satisfaction in patients requiring ABG compared to nocebo and neutral communication.

**Methods:**

A single-center, double-blind randomized controlled trial (RCT) with 3 parallel arms will be conducted with 249 patients requiring ABG during their ED visit. Patients will be randomly assigned to 1 of 3 groups before receiving ABG: positive communication group, negative communication (nocebo) group, or neutral communication (neutral) group. The communication and the words used by the physicians during hygiene preparation, artery location, and puncture will be imposed in each group. The study will be proposed to each patient corresponding to the inclusion criteria. The physicians will not be trained in hypnosis or in positive therapeutic communication. The procedure will be recorded with audio recorders to test its quality. Intention-to-treat analysis will be performed. The primary endpoint is the onset of pain. The secondary outcomes are patient comfort, patient anxiety, and global satisfaction of the patient with the communication strategy used.

**Results:**

On average, 2000 ABG procedures are performed each year in the EDs of hospitals. In this study, 249 patients are expected to be included. With a projected positive response rate of 80%, we intend to include 25 (10%) patients per month. The inclusion period began in April 2023 and will run until July 2024. We hope to publish the results of the study during the fall of 2024.

**Conclusions:**

To the best of our knowledge, this study is the first RCT assessing the use of positive communication on pain and anxiety in patients undergoing the ABG procedure in the ED. A reduction in pain, discomfort, and anxiety is expected when using positive communication. If the results are positive, this could be useful to the medical community and encourage clinicians to monitor their communication during care.

**Trial Registration:**

ClinicalTrials.gov NCT05434169; https://clinicaltrials.gov/ct2/show/NCT05434169

**International Registered Report Identifier (IRRID):**

PRR1-10.2196/42043

## Introduction

Pain, defined as a disagreeable sensory or emotional experience, is the most frequent symptom among patients admitted to the emergency department (ED). Karwowski-Soulié et al [1] estimate that this symptom can be observed in almost 80% of patients upon their arrival to the ED. Despite several international recommendations concerning pain management [2,3], pain in the context of an emergency often remains underestimated [4]. Medical procedures, such as venous or arterial blood collection, which are essential for diagnostic management and patient care, can also generate pain and a high level of stress for patients who are already weakened by their underlying condition [5]. This is known as “care-related pain” [6].

Arterial blood gas (ABG) sampling is necessary to assess the gravity of the patient’s condition and is a procedure conducted routinely in the ED. In a medium-size hospital (30,000 hospitalizations per year), approximately 1000 ABG tests are conducted annually [7]. However, arterial blood collection is generally associated with pain and patient anxiety [8].

To decrease the perception of pain linked to the collection of arterial blood, several methods have been studied, such as applying a topical anesthetic cream (eg, lidocaine, xylocaine) or administering a subcutaneous injection of lidocaine prior to the procedure [9]. Given that arterial blood collection is often performed in emergency situations and that the anesthetic cream must be applied at least 1 hour before sampling, this method is not adapted to emergency practices. In a previous study, a subcutaneous injection of lidocaine did not allow a significant reduction in pain during radial artery puncture [10]. Similarly, another study using ultrasound guidance for arterial blood sampling did not show any significant decrease in pain, the rate of immediate complications, or physician satisfaction [11].

In addition to the therapeutic tools commonly used, communication is a key element of care [12]. In routine practice, caregivers inform patients that they are going to perform a potentially painful act. However, forewarning a patient that one will perform a painful act increases the perception of pain [13]. It has also been shown that a communication strategy including the use of positive, kind, or reassuring terms (“Let me reassure you…,” “Are you comfortable?,” “Are you warm enough?”) can contribute to decreasing pain [14,15], while conversely, the choice of terms with a negative connotation (“Okay, this might sting,” “Do not panic,” “Are you in pain?,” “Are you cold?”) may increase the perception of pain and provoke a sense of discomfort [16]. In the latter case, this is known as the “nocebo” effect [15]. A communication strategy including reassuring terms, perceived by the brain as agreeable information, can reduce the pain perception. The objective of this strategy is that the painful message becomes less present as much as possible and that the patient benefits from a feeling of control. This procedure is widely used in hypnosis communication [17] and is based on a neurobiological rationale [18,19], that is, an increase in the secretion of endogenous opioids [20-25] affecting pain modulation networks [26] decreases transmission in the pain pathways [27,28], thus leading to a release of dopamine in the striatum [29]. This technique has already been shown to be of benefit in patient management [15,30] in various domains, such as anesthesia and pediatrics [15,31].

Arrival to the ED can induce a particular state of consciousness linked to anxiety and a heightened suggestibility of negative communication. Although several studies have compared the impact of verbal attitudes, notably in the domain of anesthesia and mainly with staff already trained in hypnosis, to the best of our knowledge, none have studied the effect of positive communication by caregivers or physicians among a patient population in the ED setting. Given the limited therapeutic options to relieve the pain and discomfort of blood collection performed in the ED, this study will investigate the effect of positive therapeutic communication on pain, anxiety, discomfort, and global satisfaction in patients requiring ABG compared to nocebo or neutral communication.

## Methods

### Study Design

This study will be a single-center, double-blind randomized controlled trial (RCT) with 3 parallel arms. To minimize bias during the intervention itself, the communication strategy will be clearly defined beforehand. The evaluator (any person [physician/student/other] assessing patient outcomes before and after arterial blood collection but playing no role in patient management in the ED or in the conducting of the procedure and without any knowledge of the study arm assigned to the patient at randomization) will be blinded to the strategy used.

### Setting

Patients will be recruited at the adult ED of the Cantonal Hospital of Fribourg (HFR) in Switzerland. On average, 2000 ABG procedures are performed each year in the ED of the HFR.

### Population and Sample

The inclusion criteria are as follows:

- Patients over 18 years of age
- Patients scheduled to undergo radial artery blood collection during their visit to the ED in order to pursue routine care
- Free and informed consent obtained from the patient

The exclusion criteria are as follows:

- Patients incapable of judgment
- Patients with conditions that render them unable to participate in the intervention in this study (eg, cognitive impairment, major hearing loss without the use of hearing aids, acute psychiatric disorders, under intravenous or oral sedation)
- Patients with an insufficient understanding of the French language, as defined by self-evaluation
- Patients in critical situation requiring immediate resuscitation
- Local anesthesia (subcutaneous, transdermal patch) at the point of ABG puncture
- Patients already treated with anxiolytics or sedatives during care in the ED

#### Recruitment and Screening

Potentially eligible patients for inclusion in the study will be identified by residents who will inform the senior physician responsible for triage (triage doctor [TD]) in the ED at the initial stage of patient management if arterial blood collection is required for medical care. The senior TD (triage medical consultant regulating the flow of activity in the ED, who can be a physician investigator) will proceed with recruitment as follows: “We have explained to you that we need to perform a blood gas test on a sample of blood from the wrist artery. At present, we are conducting a study to examine the patient experience with this procedure. In this context, if you accept to participate in our study, another person will ask you some questions before and after sample collection. You are free to participate and to withdraw at any point of time during the study. If you refuse, there will be no modification of your care in the ED. The data collected will be coded, and your name or any other personal information that could identify you will be removed. The entire procedure will be recorded using an audio recorder.”

The study will be proposed to all eligible individuals from among participants available 24 hours a day, 7 days a week. With a projected positive response rate of 80%, we intend to include 25 patients per month. To achieve the desired sample size, we would need 12 months to finalize study inclusion.

### Data Collection

The clinician in charge of the patients will determine the indication for ABG testing according to the rules of good practice in emergency medicine (hypotension and shock, dyspnea and respiratory insufficiency, intoxication, metabolic and acid-base disorders, renal insufficiency, polytrauma, convulsions, disorders of the state of consciousness). Patients in critical situations requiring immediate resuscitation will be excluded from the study because of the impossibility of waiting for the time necessary for screening/inclusion/randomization before ABG testing, the immediate vital prognosis being engaged.

Following verification of the inclusion criteria and patient agreement, the patient will be included in the study by the senior MRT. The latter will collect written signed informed consent forms after the procedure, which will be stored in a dedicated file cabinet and locked by 1 of the physician investigators (physicians with a role in the concept, implementation, or organization of the study). The forms will then be filed in a file reserved for this purpose and stored in a secure location by the physician acting as the principal investigator (PI).

Next, the patient will be randomized to 1 of the 3 study arms by an administrative reception agent, who will use Research Electronic Data Capture (REDCap) software (Vanderbilt University) to assign the patient to a randomization arm. To maintain the blinding of those involved in the study, the administrative reception agent will give the participating physician an opaque envelope containing randomization arm details and an audio recorder. The participating physician (resident performing arterial blood collection, who can concomitantly manage the medical aspect of the patient) will only be able to open the envelope once they are in the patient booth in order to maintain the blinding of the senior MRT. They will perform arterial blood collection according to the communication method generated by the randomization arm and will be the only person aware of the patient groups.

The senior MRT will also communicate the patient ID to the evaluator. Data collection, as well as the case report form (CRF), will be completed by the evaluator before the beginning of the recording and after completion of the procedure. Data will be collected with the help of the electronic patient record and the questions asked to the patient before and after the ABG procedure. Thus, the patient will be blinded to the specific intervention status.

### Implementation of Arterial Blood Collection

Sample collection will be performed in the patient booth. The booth door will be closed, and the shutter lowered during the intervention to allow noise and visual isolation. Only the physician performing the intervention and the patient will be in the booth during the procedure. At that time, the physician will open the envelope with their back to the patient at the same time as they prepare the material for the arterial puncture. They will then perform arterial blood collection by using 1 of the different standardized communication methods according to the group assigned to the patient: therapeutic positive communication, neutral, or nocebo. The physician assistants participating in the study have not received any training in hypnosis or in-depth instruction in positive therapeutic communication. They will carry out the intervention in all 3 study arms.

Before performing the procedure, the participating physician will switch on the audio recorder and record their conversation with the patient. The recording will start just before sampling. When the collection procedure is terminated, the participating physician will stop recording, place the text and the audio recorder in the envelope, and give it to the senior MRT. The evaluator will then come to collect the audio recorder and evaluate patient outcomes by using the CRF intended for this purpose.

Arterial blood collection will be conducted in a seated position for all 3 groups. The patient will be in a supine position, with the forearm comfortably placed on a small table. The needle used for blood collection will be a 25-gauge needle without local anesthetic. This method of sampling is identical to the one used in daily practice in the ED of the HFR.

In the event of failure of the first puncture, the physician in charge of the patient will be able to use other terms corresponding to the assigned intervention group. In the event of 2 puncture failures by the participating physician, the patient will be excluded from the study.

### Case Report Form

The following data will be collected and included in dedicated CRFs. The paper CRF will contain a first part with the date of the screening, the name of the medical officer who will perform the screening, the inclusion and exclusion criteria, whether the patient has been informed orally, and whether they have provided oral consent. The medical officer will confirm whether the inclusion and exclusion criteria have been met and, if so, indicate the number of the envelope containing the random allocation to 1 of the 3 study arms; this number will be the patient's ID in the study.

Once the patient is included in the study, the assessor will fill in the second part of the paper CRF before the ABG procedure: the name of the assessor, educational level, native language (French/Swiss German/German/Italian/Portuguese/other), tobacco, diabetes, first 3 outcomes, vitals (blood pressure, heart rate, respiratory rate), and time data on the ABG procedure.

After the ABG procedure, the evaluator will fill in the third part of the paper CRF, containing the 5 outcomes, the level of overall satisfaction, the vitals (blood pressure, heart rate, respiratory rate), the number of arterial punctures observed on the skin, and the type of words used by the doctor during the procedure according to the patient.

The fourth part of the paper CRF certifies that the patient has received the complete information about the study, whether the patient has consented, and, if they have consented, whether they have received a copy of the signed informed consent form.

The fifth part of the paper CRF contains the following information extracted directly from the patient's file: age, sex, first and last names of the attending physician, duration of the ED visit (difference between admission and discharge from the ED, difference between admission to the ED and the patient's placement in the cubicle), reason for admission to the ED (Swiss emergency triage scale [U1-U4]), list of emergency reasons, BMI (weight/(height in m × height in m), and the patient outcome after the emergency stay (inpatient/outpatient/death).

The evaluator will confirm the accuracy of the data collected.

### Intervention Groups

During each step of the sampling (identification, disinfection, sampling) for the ABG procedure, the physician involved will communicate with the patient with a preestablished speech according to the assigned study arm. Communication sentences for each intervention group are presented in Table 1. In the case of failure of the first puncture, the intervening doctor will be able to use the second set of sentences corresponding to the assigned intervention group (also in Table 1).

**Table 1:** Study arms.

Study arm	Intervention/treatment
Positive therapeutic communication: Solicit the subconscious mind of the patient by asking questions that are not focused on the ABG^a^ procedure. This process allows to divert the attention of the brain to more pleasant information and thus modulate the painful information. This is a standardized intervention with precise phrases.	The positive therapeutic communication group physician will say: During identification of the arterial blood collection site: “Are you comfortably installed?,” “How old did you say you were?,” etc At the time of disinfection: “How did you get to the hospital?,” “What day is it?,” etc At the time of puncture: “What is the noise of the lights at your home?,” “Does your car still go to the shops?,” etc
Nocebo: Use of words with a negative connotation (words used frequently in daily practice in the ED^b^)	The nocebo group physician will say: During identification of the puncture site: “That’s where I am going to prick you,” “I am looking for the place where I will prick,” etc At the time of disinfection: “Watch out, it is cold,” “It is disagreeable,” “I am disinfecting the site,” etc At the time of puncture: “Watch out! I am going to prick…1, 2, 3…I am pricking,” “Watch out, this is going to hurt, I am pricking,” etc
Neutral: Use of words defined as neutral (ie, no negative or positive connotation but based on the description of precise facts)	The neutral group physician will say: During identification of the site: “I am taking your pulse,” “I am identifying the artery,” etc At the time of disinfection: “I am disinfecting the site,” “I am cleaning your skin,” etc At the time of puncture: “I am taking the sample,” “I am performing the puncture,” etc

^a^ABG: arterial blood gas.

^b^ED: emergency department.

#### Positive Therapeutic Communication Arm

The first 2 sentences allow the gesture to be accompanied by reassuring words and positive words, while the last sentence involves the patient's subconscious by asking a question not focused on the procedure (confusion). This allows the brain to divert attention to more pleasant information and thus modulate the painful perception.

A study of a similar design, “Hypnosis and Communication Reduce Pain and Anxiety in Peripheral Intravenous Cannulation: Effect of Language and Confusion on Pain During Peripheral Intravenous Catheterization” (KTHYPE) [15], has already evaluated the impact of these sentences during the placement of a venous catheter in patients undergoing surgery and demonstrated its beneficial effects.

All the short phrases in this protocol are inspired by the KTHYPE study and have been adapted for the population and context of our study.

#### Nocebo Arm

The use of words with negative connotations would cause more pain and anxiety [13,18]. Warning a patient that they are going to undergo a potentially painful procedure can potentiate discomfort. In current practice, these are the words most used by emergency teams. The stress associated with the emergency consultation could further amplify the painful experience and the impact of these words by the state of hypersuggestibility that it induces.

#### Neutral Arm

The use of neutral terms allows precisely describing the procedure to the patient and informing them of the course of the treatment without using words with negative connotations (pain, stinging, unpleasant).

### Hypotheses

The principal hypothesis is as follows: Pain perceived during the collection of an arterial blood sample in the ED is lower when it is performed using positive therapeutic communication compared to neutral or nocebo communication.

The secondary hypotheses are as follows:

- Discomfort perceived during the collection of an arterial blood sample in the ED is lower when the procedure is conducted using positive therapeutic communication compared to neutral or nocebo communication.
- Anxiety perceived during the collection of an arterial blood sample in the ED is lower when the procedure is conducted using positive therapeutic communication compared to neutral or nocebo communication.

### Outcomes

The primary outcome is pain: self-evaluation of the maximum pain felt by the patient during the collection of an arterial blood sample on the Verbal Rating Scale (VRS: 0=no pain to 10=the worst imaginable pain) just before the beginning of the procedure and during the procedure (question asked during the 3 minutes after the procedure) [14,31,32].

The secondary outcomes are as follows:

- Comfort: self-evaluation of patient comfort using a simple comfort VRS (0=no comfort to 10=the most comfort imaginable) just before the beginning of the procedure and 3 minutes after the procedure [33]
- Anxiety: self-evaluation of patient anxiety using a simple anxiety VRS (0=no anxiety to 10=the worst anxiety imaginable) just before the beginning of the procedure and 3 minutes after the procedure [34]
- Puncture failure: success of the first arterial puncture assessed by the accredited evaluator at the end of the procedure using the number of puncture points visible on the patient’s skin (possible response: 1 puncture visible, yes/no)
- Global satisfaction with the communication strategy used: evaluation by the patient at the end of the procedure using a numeric scale of 0-10 (0=extremely dissatisfied to 10=extremely satisfied)

### Statistical Analysis

#### Sample Size Calculation

With a significance level of .05, a power of 0.9, a difference in pain on the VRS of 1.5 (generally accepted as clinically significant) with an expected SD of 2.5 [35], and an attrition of 20% on the basis of a group-independent *t* test (a specific case of linear mixed effects models [LMMs]), we must include 83 patients in each group to show the efficacy of the intervention on pain by applying the Bonferroni correction (ie, a total of 249 patients).

#### Statistical Analysis Plan

First, descriptive statistics (frequencies and percentages for categorical data; means, SDs, medians, and IQRs for quantitative data) will be run for each measure of time for each group and the overall study sample. In addition, an analysis of nonresponses and patient dropouts will be conducted. Second, bivariate associations will be calculated to compare the groups at baseline (Pearson chi-square test, Fisher exact test, *t* test, and the Mann-Whitney test according to the distribution of variables). Third, to test the impact of the intervention on the different outcomes (hypotheses 1, 2, and 3), LMMs with patients, groups and physicians as a random effect will be performed to analyze changes over time. The participation of approximately 25 physicians is foreseen in this study. The models will be tested on all available data.

The effects of time, groups, and their interaction will be tested by controlling for sociodemographic characteristics and variables linked to health status. An intention-to-treat (ITT) analysis, per protocol (PP) and based on the analysis of communications (ie, the respect of the quality of the defined communication according to the protocol; see the next section), will be conducted. The Pearson chi-square test comparing the allocation defined in the protocol and the allocation following the analysis of communications will be performed. If necessary, a generalized LMM will also be used to explore the differences between the available measured variables (physicians having performed the ABG procedure, pain, etc). Statistical analysis will be performed on available data, and no imputation of missing data will be conducted. Given that our study will be conducted on a 1-to-1 basis with a member of the research team and on the basis of previous experience, we expect that the number of missing values will be low. Statistical analyses will be performed using the R (R Core Team and the R Foundation for Statistical Computing) and STATA version 12 (StataCorp); the level of significance will be set at *P*=.05.

#### Analyses of Communications

All communications will be recorded. We will listen to 30 communications at a time to evaluate the quality of each communication and to ensure that the communication corresponds to the defined study arm defined at the time of randomization. This evaluation will be performed until less than 10% of incorrect rankings are obtained. A physician external to patient management will reassign the patient groups based on a list of preselected keywords. This same physician will then (re)classify the patients into 1 of the following 3 arms: therapeutic positive communication, nocebo, or neutral.

The classification will be performed with the help of a list of 10 keywords provided to the external physician. Each list of 10 keywords will be associated with 1 of the 3 arms and will correspond to standardized phrases related to each arm. Thus, if the external physician finds 1 keyword associated with the nocebo arm, the patient will then be reclassified into the nocebo arm; if the external physician finds 1 keyword associated with the therapeutic positive communication arm but without any keywords associated with the nocebo arm, the patient will be classified into the therapeutic positive communication arm. If the physician does not find any keywords associated with these 2 arms, the patient will be classified into the neutral arm.

This classification will be conducted in parallel by 2 persons for the first 30 patients to evaluate the interrater reliability using Cohen κ. If κ>0.90, only 1 coder will continue to code the audio recordings. If not, 2 coders will continue to evaluate the communications; in the case of disagreement, a third coder will make the final decision.

The keywords associated with the nocebo group are as follows:

- Pain
- Cold
- Prick
- Do not panic.
- Are you in pain? I am going to prick.
- This is going to sting.
- Do not move, it won’t be long.
- Do not be frightened.
- How do you feel?

The keywords associated with the therapeutic positive communication group are as follows:

- Be reassured.
- Are you comfortable?
- Are you relieved?
- I am going to place the drip.
- Have confidence.
- Take it easy.
- It will be fast.
- Do you feel well?

These lists are likely to be adapted based on the audio recordings.

### Ethical Considerations

#### Research Ethics Approval

The study protocol was approved by the Human Research Ethics Committee of the Canton of Vaud, Switzerland (no. 2022-00685).

All participants will receive an information letter presenting the study, its objectives, and its risks. Sufficient information will be provided to allow an informed decision to be made concerning their participation in the study. The senior TD responsible for recruitment will be at the disposal of patients to respond to any eventual questions that may arise. Given the specific context of medical care in the emergency setting, a reflection time of 15 minutes will be allocated to allow each participant to make a decision. Particular focus will be given to the primacy of the medical indication for arterial blood sampling and to the fact that this study is solely concerned with the patient perception of this procedure. In this context, we consider that the time of reflection is sufficient to allow free and informed consent. Following signature of the consent form and verification of the eligibility criteria, the patient will be included in the study. As the patient will be blinded to the intervention, the information letter will only explain our hypothesis concerning the importance of communication on the emotional level during a care episode, and no information will be provided on the 3 verbal attitudes or any other details, which might influence the patient’s judgement.

In the event of the inability of a patient to provide informed consent in writing before the ABG procedure (eg, severe respiratory distress preventing them to write), verbal consent will be sufficient before inclusion and will be notified in writing on a consent sheet by the senior TD. Written consent will then be requested later after emergency care.

#### Data Monitoring

No data related to the research foreseen will be obtained via laboratory tests or invasive procedures. The sources of materials are as follows:

- Data of quantitative interviews, heteroadministered by the evaluator using a paper CRF. The data will then be entered into REDCap.
- Patient screening data collected by the senior TD.
- Data of audio recordings collected during the ABG procedure with the use of an audio recorder. Recordings will be transcribed for analysis in Microsoft Word.
- Patient information letters as well as the consent forms to be completed will be stored in a dedicated secure box for the study in the ED.

As this is a low-risk study, the following monitoring activities will be carried out by a person independent of the study and trained in good clinical practices and the protocol:

- A qualification visit will be conducted prior to the start of the study.
- The initiation visit will be conducted in the HFR ED in the presence of the PI and the research team.
- A follow-up visit after the inclusion of the first 3 participants will be carried out and if necessary repeated in the case of major problems encountered.
- Regular monitoring (minimum once every 3 months) will be carried out to (1) check that all patients have provided oral consent before inclusion and written consent after the procedure, (2) check that documents are stored according to the protocol (consents, paper CRFs, etc), (3) verify paper CRF data extracted from the patient records (at least 20% of data randomly selected), (4) verify the concordance of the information in REDCap and the information in the paper CRFs (at least 20% of the randomly selected data), and (5) verify the adequacy of the caregivers' communication with the allocation arm for the first 30 patients included. Control visits will be conducted if the actual communication differs from the allocation arm in more than 10% of cases.

#### Data Confidentiality

Data concerning the trial and the participants will be treated with the greatest-possible confidentiality and will only be accessible to authorized staff who need the data to fulfil their role within the context of the study. Participants will only be identified on the CRFs and other specific study documents by their unique IDs.

The production, transmission, storage, and analysis of personal health data in the context of this study will strictly respect current Swiss legal requirements concerning data protection and will be conducted in conformity with Ordonnance ClinO Art. 18. All lists of names (or other identifying information), personal IDs, and consent forms will be stored in a locked office at the HFR in the offices of the PI. Access to the initial lists and signed informed consent forms will be limited to the physician investigators (authors CLT, YG, and TS).

All electronic data will be recorded using only personal IDs in REDCap.

Quantitative interview data will be collected using paper CRFs. All participants will be identified only by study IDs. The list corresponding to identifying information and the study IDs will be stored in a file protected by a password and only be accessed by the physician investigators.

Data of physician communication during the ABG procedure will be recorded using a numeric recording machine. Audio files will be labelled only with the ID number and transferred to computers protected by a password to be stored electronically. Interviews will be transcribed in Word files containing only the ID number and then stored electronically on computers protected by a password and on secure servers at HFR. Following verification of the transcripts, the audio files will be erased.

### Retention and Destruction of Study Data

All the study data will be archived for 10 years after termination of the study or premature termination. The signed informed consent forms will be kept at the HFR and immediately placed in secure locked cupboards. All paperwork (including printed data) will be archived for a minimum of 10 years after the end of the study in locked cupboards at the HFR. Participants who withdraw their consent or express the wish to end the research interview will be excluded from the study. Data collected until that time will be analyzed so as not to compromise the validity of the study, unless they request the deletion of their data.

## Results

On average, 2000 ABG procedures are performed each year in the ED of the HFR. Recruitment for this RCT began in April 2023. In total, 249 patients are expected to be included. The inclusion period will run from April 2023 to July 2024.

## Discussion

### Summary

To the best of our knowledge, this is the first RCT that will investigate the effect of a positive communication over a routine procedure, such as ABG testing, in an emergency setting. Considering the KTHYPE study [15], a reduction in pain, anxiety, and discomfort can be expected when using positive communication.

Two of the ethical values in the medical field are beneficence and the objective to do no harm to the patient. However, although the nocebo effect of language is acknowledged today in specialties, such as anesthesiology, negative communication continues to be used daily in the ED, a particularly stressful setting with vulnerable patients. This makes it all the more important to study language tools in this environment, with the aim of standardizing and protocolizing caregivers' communication.

ABG testing is an investigation required in certain medical situations. The global experience of this procedure has often been described as disagreeable and can be improved [8]. The comfort and anxiety before and after sampling can be demonstrated. Today, no risk linked to the communication strategy has been documented, except for the usual inherent risks of arterial blood collection. The nocebo arm is nowadays commonly used in routine practice. If communication can lead to decreased pain, anxiety, and discomfort during this standard investigation, it will lead to changes in current practice. This method is easily used by even the most inexperienced doctors, and we hope that this will reduce their stress during the procedures.

Our study will also assess patient comfort and anxiety. These elements are of primary importance, as pain is frequent in emergencies and frequently underestimated (80% of patients [1]). The patient's anxiety and comfort level contribute to this pain, even if it has not been well documented. To interpret the results, we may compare the comfort and anxiety scores before and after the procedure. This will allow us to control for patients with chronic pathologies (eg, depression).

### Strengths and Limitations

The main strengths of the study are its originality, the ease of implementation, and the rigorous methodological design, the last being based on a previously published study [15].

However, this study also has some limitations. First, the received communication policy may be different from the one intended to be used. To limit this bias and avoid any contamination of the arms due to inappropriate communication, all procedures will be recorded and replayed to verify whether the attributed group and the received communication scheme correspond. Second, this is a single-center study. The experience of the physicians performing the ABG procedure, although similar, is quite limited, which could interfere with the communication, as these inexperienced physicians may focus more on the correct technical execution of the ABG procedure than on the communication. In addition, nonverbal communication during the interview will not be studied. Third, other uncontrolled confusing factors could affect the results (eg, a long wait in the waiting room for the patient and overloaded service for the doctor), but we hypothesize that these factors may be similar in each group due to randomization and will thus limit the potential bias.

### Conclusion

The Positive Therapeutic Communication on Pain (POPAIN) trial is an RCT assessing the effect of positive communication on pain, discomfort, and anxiety in patients undergoing ABG testing compared to neutral and nocebo communication. A reduction in pain, discomfort, and anxiety is expected when using positive communication. These results could be useful to the medical community and encourage clinicians to monitor their communication to reduce pain and anxiety in care. This method, if successful, could be easily implemented in other emergency departments.

## Abbreviations

ABG: arterial blood gas
CRF: case report form
ED: emergency department
HFR: Cantonal Hospital of Fribourg
KTHYPE: Hypnosis and Communication Reduce Pain and Anxiety in Peripheral Intravenous Cannulation: Effect of Language and Confusion on Pain During Peripheral Intravenous Catheterization
LMM: linear mixed effects model
PI: principal investigator
RCT: randomized controlled trial
REDCap: Research Electronic Data Capture
TD: triage doctor
VRS: Verbal Rating Scale

## Data Availability

Once data are collected and ready for publication, they will be comprehensively presented in the manuscript. The results will be distributed in national and international scientific conferences and medical peer-reviewed journals.
